# Drought increases root and rhizodeposition carbon inputs into soils

**DOI:** 10.1007/s11104-025-08021-1

**Published:** 2026-01-12

**Authors:** Elena Kost, Dominika Kundel, Matti Barthel, Rafaela Feola Conz, Roland Anton Werner, Shiva Ghiasi, Tabata Aline Bublitz, Paul Mäder, Hans-Martin Krause, Johan Six, Martin Hartmann, Jochen Mayer

**Affiliations:** 1 Institute of Agricultural Sciences, Department of Environmental Systems Science, ETH Zürich, Zurich, Switzerland; 2 Departement of Soil Science, Research Institute of Organic Agriculture, Frick, Switzerland; 3 Water Protection and Nutrient Flows, Department of Agroecology and Environment, Agroscope, Zurich Switzerland; 4 Department of Soil Biology and Plant Nutrition, Faculty of Organic Agricultural Sciences, University of Kassel, Kassel, Germany

**Keywords:** Drought, Rhizodeposition, Fine roots, Roots, Belowground carbon, Cropping system

## Abstract

**Aims:**

Increasing droughts affect crop yield and health. Plants can respond to drought by adapting their root biomass, root morphology, and quality and quantity of rhizodeposition to improve water and nutrient uptake. Besides droughts, agricultural management influences roots and rhizodeposition; however, it is not well studied how agricultural management can affect the response of roots and rhizodeposition to drought.

**Methods:**

A semi-continuous ^13^CO_2_ isotope labelling experiment was performed in a long-term field experiment comparing biodynamic, mixed conventional, and mineral conventional cropping systems. Rainout shelters were installed to induce drought. Root, net rhizodeposition, and the rhizosphere microbiome were determined at ripening of wheat.

**Results:**

Drought enhanced the total root carbon mainly through the increase of fine roots. Fine root carbon under drought was primarily enhanced in the mixed conventional and biodynamic cropping system, both receiving farmyard manure, whereas no increase was measured in the mineral fertilized conventional system. Net rhizodeposition carbon was enhanced in all cropping systems under drought, particularly in the first 0.25 m. While some plant-growth-promoting genera such as *Streptomyces* and *Rhizophagus* showed relative increases under drought, other plant growth-promoting genera often involved in nitrogen fixation such as *Rhodoferax* and *Mesorhizobium* were decreased.

**Conclusion:**

This field trial suggests that drought increases total belowground carbon input via fine root and net rhizodeposition carbon inputs. Since fine root carbon increased under drought in cropping systems with farmyard manure, adding manure under future drought periods could be advantageous to increase soil carbon inputs and improve nutrient foraging.

**Supplementary Information:**

The online version contains supplementary material available at 10.1007/s11104-025-08021-1.

## Introduction

As a consequence of climate change, droughts are becoming more frequent and severe, which can adversely affect crop health and growth (Lesk et al. 2016; IPCC 2023). Droughts are estimated to be a major threat to global crop production (Lesk et al. 2016). Plants can alter their root biomass and morphology as well as the quantity and quality of rhizodeposition in response to drought (Steinemann et al. 2015; Liang et al. 2017; Djanaguiraman et al. 2019; Williams and de Vries 2020). Both factors are essential for plant water and nutrient uptake (Oren and Sheriff 1995; Bais et al. 2006), which are especially limited under drought. Rhizodeposition mainly consist of low molecular weight solutes (e.g. sugars, amino acids, and organic acids), high molecular weight polysaccharides (mucilage), border cells, root caps, and dead roots (Jones et al. 2009). They can increase soil nutrient and water availability for plants by affecting soil chemistry and biology (Bais et al. 2006).

Some studies show a reduction in root biomass, length density, and thickness under drought (Hutchings and John 2003; Rich and Watt 2013; Steinemann et al. 2015; Liang et al. 2017; Djanaguiraman et al. 2019), yet it depends on plant species, drought severity, and duration (Hutchings and John 2003; Comas et al. 2013; Zhou et al. 2018; Xiao et al. 2020). Besides drought, the type of cropping systems can also influence the rooting systems. Recent studies showed increased absolute and relative root carbon (C) allocation in organic and low-fertilized systems, particularly in the topsoil (Chirinda et al. 2012; Chowdhury et al. 2014; Hu et al. 2018; Hirte et al. 2021), while other studies reported no effect of the cropping system on the root biomass and length (Steingrobe et al. 2001; Hirte et al. 2018a). Roots are not only important for water and nutrient uptake, but root C inputs are also considered to be important for mid and long-term C storage (Poeplau et al. 2021). While many studies assess the individual effects of drought or cropping systems on roots, the impact of cropping systems in the context of drought stress is not well studied.

Conventional and organic cropping systems differ in many soil characteristics, including but not limited to soil nutrient content, organic C content, microbial composition, aggregate formation, and enzyme activities (Mäder et al. 2002; Hartmann et al. 2015; Krause et al. 2022). These differences result from contrasting agricultural practices (i.e. crop rotation, fertilization, pesticide, and plant growth regulator application), which might affect how biomass and characteristics of roots and rhizodeposition respond to drought.

Around 15–30% of net assimilated C by cereals is allocated belowground, of which up to 65% can occur as rhizodeposition in the soil (Swinnen et al. 1994a; Kuzyakov and Domanski 2000; Hirte et al. 2018a). Rhizodeposition are considered important for C sequestration, plant immunity, plant–microbe interactions, soil aggregation, soil detoxification, and nutrient uptake, as they deliver root-derived compounds such as exudates, mucilage, and shedded cells that fuel microbial activity, bind soil particles, chelate toxins, and mobilize nutrients (Bais et al. 2006; Villarino et al. 2021). Rhizodeposition can change in quality and quantity in response to drought, with either an increase of rhizodeposition per gram plant biomass under moderate drought, while showing variable effects under extreme drought (Preece and Peñuelas 2016; Williams and de Vries 2020). Some studies have shown that agricultural practices like fertilization intensity can increase total C rhizodeposition (Liljeroth et al. 1994; Swinnen 1994; Qiao et al. 2017), whereas others suggest no effect of fertilization intensity on total C rhizodeposition (Hirte et al. 2018a). Organic fertilization might enhance total C rhizodeposition, possibly by stimulating root exudation through improved nutrient availability or by reducing microbial mineralisation of rhizodeposited C due to increased supply of C through organic fertilization (Hirte et al. 2018a). Soil physical and chemical properties such as porosity, water retention, organic matter content, and pH can differ between conventional and organic cropping systems (Mäder et al. 2002; Krause et al. 2022) and influence rhizodeposition by affecting root growth, exudation rates, and microbial turnover (Van Veen et al. 1989; Pausch and Kuzyakov 2018). For example, acidity and temperature can increase exudation (Lynch and de Leij 2012). Moreover, organically fertilized systems might sustain better root growth and rhizodeposition under drought because of improved soil structure and moisture retention (Mäder et al. 2002). Under drought, roots may increase root exudation to mobilize nutrients from organic matter via priming and enzymatic mineralization, processes that might be enhanced in organically fertilized soils with a higher organic matter content (Krause et al. 2022). For instance, nutrient availability may be more limited in organically fertilized systems, especially under drought, leading to increased root foraging and exudation activity (Hirte et al. 2018a, 2021) compared to mineral-fertilized systems. It is not well understood how cropping systems differing in fertilization affect rhizodeposition under drought.

Drought also affects soil microbes (Naylor and Coleman-Derr 2018; Ochoa-Hueso et al. 2018). Soil microbes are crucial for soil functioning and crop production, as they are involved in nutrient cycling, climate regulation, pollutant degradation, plant growth promotion and stress tolerance, and disease and pest control (Hartmann and Six 2023). Rhizodeposition is an important link between plants and soil microbes for communication (Williams and de Vries 2020) and a C-source for microbes (Bais et al. 2006; Dennis et al. 2010), particularly in the rhizosphere, defined as the soil around the roots under the influence of living roots (Edwards et al. 2015). Rhizosphere microbiomes, which are more closely associated with roots, have been found to be more strongly affected by drought compared to bulk soil communities (Naylor et al. 2017; Santos-Medellín et al. 2017). This is possibly in part due to rhizodeposition (Jones et al. 2009), which changes in quantity and quality due to drought (Williams and de Vries 2020). Through rhizodeposition, plants are able to recruit microbes that improve plant drought tolerance by, for example, increasing their exudation of organic acids such as malic acid to attract *Bacillus subtilis* or glycerol-3-phosphate, an important precursor to peptidoglycan biosynthesis, to support growth of gram-positive bacteria (Hartman and Tringe 2019; Williams and de Vries 2020). Plant growth-promoting (PGP) microbes can enhance plant tolerance to drought by increasing plant osmolyte content, nutrient uptake, abscisic acid, or auxin concentrations, production of exopolymeric substances, and decreasing plant ethylene (Ortiz et al. 2015; Ngumbi and Kloepper 2016; Lata et al. 2018). Some bacteria, categorised as slow-growing oligotrophic bacteria, are considered to grow well under nutrient-poor conditions and are potentially better adapted to water-limited conditions, as opposed to copiotrophic bacteria, which flourish under nutrient-rich and well-watered conditions (Naylor and Coleman-Derr 2018).

In this study, we assessed the effects of severe drought on root C content, rhizodeposition C, and the rhizosphere microbiome of winter wheat (*Triticum aestivum* L.*)* at ripening in the long-term field trial DOK in Switzerland, comparing organic and conventional management practices since 1978. The systems differ in their application of fertilizers, pesticides, and plant growth regulators. Our study focused on net C rhizodeposition, which is defined as C remaining in the soil after microbial consumption and reuptake of previously released compounds by roots (Pausch and Kuzyakov 2018). A semi-continuous pulse labelling experiment was conducted using ^13^C-CO_2_ in a field-scale drought simulation. Based on the current literature, we hypothesized that (1) the allocation of total belowground C (hereafter defined as fine and coarse roots and rhizodeposition C) will increase under drought for all treatments, (2) belowground C under drought will increase more under sole organic fertilization compared to cropping system that combine organic and mineral fertilization or are exclusively mineral fertilized and, (3) bacterial and fungal taxa known to enhance plant drought tolerance such as those involved in phytohormone production, stress hormone modulation, nutrient mobilization, and biocontrol will increase in the rhizosphere in response to drought.

## Methods

### Experimental setup

A field-scale drought simulation was performed in the DOK long-term field trial situated in Therwil, Switzerland (47°30′9.48"N, 7°32′22.02"E; 330 m a.s.l.) from mid-November 2021 to the beginning of July 2022. The DOK trial compares organic and conventional cropping systems since 1978 and has been described in more detail by Krause et al. (2022). Briefly, the site has an average annual precipitation of 840 mm, a mean annual temperature of 11 °C, and is located on a Haplic Luvisol from Loess deposits.

After sowing winter wheat (*Triticum aestivum* var. Wiwa) in mid-October 2021, steel cylinders with a diameter of 0.35 m and a depth of 0.30 m were installed in the soil down to 0.25 m depth (hereafter called microplot). Subsequently, rainout shelters were installed as described in Kost et al. (2024) in mid-November 2021 in three cropping systems: biodynamic (BIODYN), conventional mixed systems (CONFYM), and mineral fertilized conventional system (CONMIN). These three cropping systems differ in fertilization and pesticide management but not in crop rotation and tillage (Table A1) (Krause et al. 2022). CONFYM and CONMIN receive synthetic pesticides and plant growth regulators (chlormequat chloride and trinexapac-ethyl) according to Swiss regulations (Federal Department of Economic Affairs Education and Research 2023). CONMIN is fertilized exclusively with mineral fertilizer, while CONFYM receives a combination of organic (stacked manure and slurry) and mineral fertilizer. BIODYN is solely fertilized by organic fertilizers, including composted farmyard manure and slurry. Additionally, biodynamic preparations and no synthetic pesticides are applied according to the regulations of Demeter Schweiz (Demeter Schweiz 2019). Manure-based cropping systems (CONFYM and BIODYN) represent mixed crop-livestock systems and are supplied with cattle manure according to 1.4 livestock units per hectare annually. The number of wheat plants in December 2021 was homogenized to 35 ± 2 plants within each microplot. The drought-induced plots were irrigated either with precipitation water collected in water tanks or tap water (when tanks were empty) during winter for a total of 55 mm. From April, these plots were deprived completely of water until 14 July 2022. More details on the irrigation plan can be found in Kost et al. (2024). All agricultural management activities of the wheat season 2021/2022 are listed in Table A1.

Air temperature, photosynthetic active radiation, and soil temperature were monitored using HOBO (EnviroMonitors, Arundel, United Kingdom), PAR Photon Flux Sensors (METER Group, Pullman, WA, USA), and time domain reflectometry soil sensors (TDRs; METER Group) as described in Kost et al. (2024). An increase of air and soil temperatures by 0.4–1.2 °C and 1.1–1.6 °C below the shelters was measured, respectively (Kost et al. 2024). It is expected that increasing drought conditions will not only result from reduced precipitation but also from increased evapotranspiration caused by higher temperatures (Dai 2013; Spinoni et al. 2018). Since the start of the field trial in 1978, the mean annual temperature has increased from 9.7 °C to 10.9 °C (Krause et al. 2022), and will very likely continue to rise (IPCC 2023). Hence, rainout shelters represent climate change conditions also in terms of higher temperatures. The photosynthetic active radiation was reduced due to sheltering to an average of 870 μmol m^−2^ s^−1^ compared to 1290 μmol m^−2^ s^−1^ in the control (Kost et al. 2024). Since the light saturation of wheat for C assimilation is around 1000–1200 μmol m^−2^ s^−1^ (Cabrera-Bosquet et al. 2009; Pang et al. 2018), this might have reduced the C assimilation below the shelters. However, since the photosynthetic active radiation was close to light saturation, the reduction of C assimilation caused by the sheltering is likely small (Cabrera-Bosquet et al. 2009; Pang et al. 2018).

### Stable isotope labelling campaign

Since single pulse labelling is not suitable for studying net rhizodeposition C over an entire growing season (Kuzyakov and Domanski 2000) and continuous labelling is not feasible in a field experiment, we used a semi-continuous multi-pulse labelling approach (Keith et al. 1986; Swinnen et al. 1995; Martens et al. 2009; Hirte et al. 2018a). Multi pulse labelling with ^13^C-CO_2_ was performed during the most active C-assimilation phase from stem elongation to ripening (Fig. A1) (Kuzyakov and Domanski 2000; Hirte et al. 2018a). Following Hirte et al. (2018a), wheat plants in microplots were labelled weekly between morning and noon for around 3 h using height-adjustable Plexiglas®-chambers (0.40 m diameter; Fig. A1). On the soil surface, the gap between the chambers and the soil was sealed with soil before labelling. Between 60–720 mL of ^13^C-CO_2_ (99 atom-%; Merck, Darmstadt, Germany) was injected in 1–12 doses during each labelling event with 60 mL syringes (HSW, Tuttlingen, Germany) at eleven timepoints during the wheat growth period (Table A1). The number of doses was determined depending on the CO_2_ concentration inside the chamber, which was monitored using a portable infrared CO_2_ analyser (LI-840A, LI-COR Biosciences, Lincoln, NE, United States). Doses were injected if the CO_2_ concentration in the chambers dropped below 150 ppm, aiming to reach 800 ppm. All chambers received the same amount of ^13^C-CO_2_ at each labelling event. At the end of each labelling event, a pulse of ^12^C-CO_2_ was injected to allow a complete tracer uptake and avoid cross-contamination of neighbouring crops after removal of the labelling chambers.

### Sampling

Sampling was done at wheat ripening at the end of June 2022 at Zadok growth stage 93 for soil and plant C isotopic composition and biological molecular analysis. Plant height, straw, fresh biomass, disease, and animal damage (i.e. counting eaten ears from tillers) were recorded in the field. Aboveground plant parts were separated into shoots/spelts (hereafter referred as straw) and grains. Dry biomass was measured after drying at 40 °C to constant weight. Roots and soil were sampled in three layers: top (0–0.25 m), intermediate (0.25–0.5 m), and deep (0.5–0.75 m). First, crown roots (i.e. thick root part directly below the shoot, including attached roots, see Fig. A2) were harvested with a shovel. Equal amounts of crown roots at similar locations in each sample with adhering soil were cut for molecular analysis of the rhizosphere and stored at −20 °C. The remaining crown roots were stored at 4 °C for C analysis. After, the remaining topsoil layer was sampled as whole monoliths, homogenized, and subsampled (around 3 kg, ~ 10% of soil mass; Fig. A2). From the intermediate and deep soil layers, two soil cores (Riverside auger, 50 mm diameter; Royal Eijkelkamps, Giesbeek, Netherlands) were taken within and between crop rows (total *n* = 4; Fig. A2). Soil weights of the intermediate and deep layers were recorded, each sample was divided into two sub-samples for molecular and C analysis (minimum 1 kg each). Unlabelled aboveground plant parts, roots, and soil samples were taken at a distance of at least 2 m from the microplot in all treatments (n = 6). All samples for C analysis were stored at 4 °C for a maximum of six weeks. Samples for microbiome molecular analysis and gravimetric water content (GWC) were stored at −20 °C until further processing.

### Root and rhizosphere processing

Soil and roots were separated according to the protocol of Hirte et al. (2018a) in an adapted three-step procedure (Fig. A3). Step 1: The harvested crown roots were first cleaned of aboveground residues and shaken over a bowl to collect adhering soil. The collected aboveground plant parts from these samples were then added to the straw biomass. The collected soil falling off the crown roots was added to the field-fresh bulk soil sample of the respective 0–0.25 m soil sample. In total, around 1 kg of homogenized bulk soil sample was sieved to 2 mm. The remaining roots were cleaned from mineral and organic residues by hand using tap water and tweezers and dried at 40 °C. Intermediate and deep soil samples were identically processed but without crown roots. Step 2: A subsample of 750 g of the 2 mm sieved field-fresh soil was mixed with deionized water (volume ratio 1:1) on an overhead shaker for 20 min and the suspension was sieved through a 0.5 mm sieve. The residues remaining in the sieve were washed with tap water and separated from organic and mineral residues by repeated decantation and collection by tweezers. Roots were collected and the 0.5 mm sieved soil was discarded. Step 3: For soil collection, a subsample of 150 g of the 2 mm sieved field-fresh soil was mixed with deionized water (volume ratio: 1:1) and shaken on an overhead shaker for 20 min. The suspension was 0.5 mm sieved and the sieve was rinsed with 400–500 mL of deionized water while soil aggregates were carefully broken down through the mesh with a rubber spatula. Sieve residues were discarded. The 0.5 mm sieved soil was collected in a glass dish and spiked with 0.5 mL 4.5 mM silver solution (redispersed poly-vinylpyrrolidone-coated nanopowder, particle size < 100 nm, Merck) to stop microbial activity (Gajjar et al. 2009; Swarnavalli et al. 2011). Roots and soil were dried at 40 °C and 80 °C until constant weight, respectively. The weight of dried root samples was recorded. Unlabelled samples were processed identically but separately. Soil was dried at 105 ◦C until constant weight to assess the GWC.

### Measurements of carbon concentrations and isotopic composition

For C and N concentration and isotopic composition analysis, grain, straw, crown, and coarse roots, and soil samples were shredded, thoroughly homogenized, and a subsample was ground using a ball mill (MM 200, Retsch, Haan, Germany). Fine roots were milled in case there was enough material or otherwise cut into small pieces with scissors and homogenized. Grain, straw, soil, and coarse and fine root samples were analysed for total C and δ^13^C with a Flash EA 1112 Series elemental analyser (Thermo Italy, former CE Instruments, Rhodano, Italy) coupled to a Delta^plus^ XP IRMS (Finnigan MAT, Bremen, Germany) via a ConFlo III (Werner et al. 1999; Werner and Brand 2001; Brooks et al. 2003). All analyses were done separately for natural abundance samples. Laboratory standards (acetanilide, caffeine, tyrosine) are referenced to the corresponding international materials provided by the IAEA (Vienna, Austria). Since the field site is practically free of carbonates, the total measured C is considered organic C (Krause et al. 2022).

### Calculations

#### Excess ^13^C calculations

The mass of ^13^C excess (*m*^*E*^(^13^C)) of the straw, roots, and soil was calculated following Van de Broek et al. (2020) calculating the atom fraction (*χ)* and atom excess (*χ*^*E*^) based on Coplen (2011).

$${m}^{E}({}^{13}C)= \frac{{\chi }^{E}\left({}^{13}C\right)*m\left(C\right)*M\left({}^{13}C\right)}{\chi \left({}^{12}C\right)*M\left({}^{12}C\right)+ \chi \left({}^{13}C\right)*M\left({}^{13}C\right)}$$

where *m*^*E*^ (^13^C) is the mass of the recovered ^13^C excess (g m^−2^), *χ*^*E*^ (^13^C) the atom excess faction (unitless), *m* (C) is the total C mass (g m^−2^), *χ* (^12^C) and *χ* (^13^C) are the ^12^C and ^13^C atom fractions (unitless), respectively and *M* (^12^C) and *M* (^13^C) are the molar weights of ^12^C and ^13^C (g mol^−1^), respectively. The respective natural abundance samples were used from the corresponding treatment and soil layers to calculate the atom excess (*χ*^*E*^) of the straw, roots, and soil.

#### Calculation of root C

To obtain the root C, the root biomass was multiplied by the measured C concentration. The fine root C in the 0–0.25 m soil layer was corrected for the proportion of extraneous organic matter (EOM; i.e. remains of organic amendments, aboveground residues, soil fauna, and root of preceding crops or weeds) according to Hirte et al. (2017).

$${f}_{RBC}=\frac{{\chi }^{E}({}^{13}{C}_{S})- {\chi }^{E}({}^{13}{C}_{EOM})}{{\chi }^{E}({}^{13}{C}_{RB})- {\chi }^{E}({}^{13}{C}_{EOM})}$$

where *f*_*RBC*_ is the root C mass fraction of the total C of the fine root sample and *χ*^*E*^ (^13^C_s_), *χ*^*E*^ (^13^C_RB_), and *χ*^*E*^ (^13^C_EOM_) are the atom excess fractions (unitless) of the total sample, clean root, and EOM, respectively. The δ^13^C_EOM_ was considered similar as measured in a prior study performed in the same field (i.e. − 29.3‰) (Hirte et al. 2017).

Root C (g kg^.1^ dry soil) was extrapolated to soil surface area for each soil layer and each root size (i.e. fine and coarse) and position (i.e. between and within the row).

$${RC}_{S}= {RC}_{M}* \rho *z$$

where *RC*_*S*_ and *RC*_*M*_ are the surface (g m^−2^) and mass-related root C (g kg^−1^ dry soil), respectively, *ρ* is the soil bulk density (kg m^−3^) and *z* is the height (m) of the sampled soil layer. The C of crown and coarse roots of the topsoil layer (i.e. 0–0.25 m) were summed. Root C of the within and between rows in the intermediate and deepest soil layer were averaged by layer as mentioned in Hirte et al. (2018b).

#### Calculation of net rhizodeposition and relative C allocation coefficient

The net rhizodeposition was calculated based on the tracer mass balance approach provided by Rasmussen et al. (2019) by determining the C derived from rhizodeposition (%CdfR).

$$\%CdfR= \frac{{m}^{E}({}^{13}{C)}_{soil}}{{m}^{E}({}^{13}{C)}_{soil}+ {m}^{E}({}^{13}{C)}_{root}}*100$$

where *m*^*E*^ (^13^C)_soil_ and *m*^*E*^ (^13^C)_root_ are the mass ^13^C excess of the soil and root (including crown, coarse, and fine root mass ^13^C excess for 0–25 cm and coarse, and fine root mass ^13^C excess for 25–75 cm), respectively. Based on the *%CdfR*, the quantity of C derived from rhizodeposition (*qCdfR*) was calculated.

$$qCdfR=\frac{\%CdfR*{RC}_{s}}{100-\%CdfR}$$

where *RC*_*s*_ is the respective soil layer's corrected total root C value. The calculation of the rhizodeposition builds on two assumptions. First, the ^13^C enrichment of the roots is homogeneous, and second, the ^13^C enrichment of roots and rhizodeposition is the same. The net rhizodeposition is defined in this paper as all compounds smaller than 0.5 mm, which also includes fine roots and root fragments smaller than 0.5 mm, as reported previously in Wichern et al. (2008).

Rhizodeposition relative to the total belowground C was calculated by dividing rhizodeposition C by the sum of root and rhizodeposition C. The relative C allocation coefficient, expressed as the proportion of whole plant C, for wheat straw, grain, root, and rhizodeposition C was calculated based on Bolinder et al. (2007). Belowground C to aboveground C ratio was calculated by dividing root C and rhizodeposition C (i.e. belowground C) by straw C and grain C (aboveground C), whereas the root C to shoot C ratio was calculated by dividing root C by straw C.

### DNA extraction, sequencing, bioinformatics, and quantitative real-time PCR

Root parts with adhering soil sampled for molecular analysis were mixed with a 30 mL buffer solution containing 6.75 g KH_2_PO_4_ and 8.75 g K_2_HPO_4_ in 1000 mL deionized water, adding 200 μL Tween® 20 (Sigma-Aldrich, St. Louis, MO, US) after autoclaving. After vortexing for 2 min, roots were separated from the rhizosphere soil using tweezers and sieving through a sterilised stainless steel 2 mm mesh. The rhizosphere soil in the residual buffer solution was centrifuged for 10 min at 4 °C with 4700 × g, decanted, and then stored at −20 °C.

DNA was extracted with the DNeasy PowerSoil Pro Kit (Qiagen, Hilden, Germany) following the manufacturer’s protocol from 0.25 g rhizosphere soil on the QIAcube Connect (Qiagen) including blanks. Purity and concentration of DNA were measured via UV/VIS spectrophotometry on a QIAxpert instrument (Qiagen) and normalized to 10 ng μL^−1^. Prokaryotic (i.e. bacterial and archaeal; V3 and V4 regions of the 16S rRNA gene) and fungal (ITS2 region of the rrn operon) markers were used for PCR amplification using the primers 341F/806R for prokaryotes and 5.85-Fung/ITS4-Fung for fungi applying the conditions described in Table A2. Three technical replicates were pooled in equal volumes and sent to the Functional Genomics Center Zurich (FGCZ, Zurich, Switzerland) for the indexing PCR. The indexed PCR products were purified, quantified, and pooled in equimolar ratios prior to pre-sequencing sequencing on the Illumina MiniSeq platform (Illumina Inc., San Diego, CA, USA) to improve optimal equimolarity across samples before the final sequencing using the v3 chemistry (PE300) on the Illumina MiSeq platform (Illumina Inc.).

A customized pipeline was employed as mentioned in Longepierre et al. (2021) including primer trimming, PhiX filtering, paired-end read merging, quality filtration, dereplication, delineation into amplicon sequence variants (ASVs), chimera removal, target verification, read mapping and taxonomically classification against the SILVA v138.1 for prokaryotic sequences (Pruesse et al. 2007) and the UNITE v9.0 fungal sequences (Abarenkov et al. 2010). The total read number was 5′082′888 (70′595 ± 7505 per sample) for 16S rRNA and 3′279′424 (46′189 ± 1694 per sample) for ITS sequences, respectively. These sequences were assigned after quality control and taxonomic assignment to 42′108 and 3801 ASVs for prokaryotes and fungi, respectively. Prokaryotic ASVs were categorized in oligotrophic and copiotrophic lifestyles using the rrn gene copy numbers on the lowest assigned taxonomic rank applying rrnDB v5.8 (Stoddard et al. 2015) and using the thresholds of ≥ 5 for copiotrophs and < 5 for oligotrophs (Bledsoe et al. 2020).

A SRBR® Green-based quantitative PCR method was applied to measure prokaryotic and fungal abundance targeting the 16S and 18S rRNA gene, respectively. An amplification inhibition test (i.e. caused by unintentional co-extraction of contaminants), standard curves from purified PCR products using a serial dilution, and qPCR amplification in technical triplicates were performed as described in Jaeger et al. (2023) using the PCR conditions listed in Table A2. The efficiency of qPCR was around 96–97% for 16S and 82–83% for 18S with R^2^ > 0.99.

### Statistics

The R version v4.4.0 (R Core Team 2024) and R Studio Version v2024.04.0 + 735 (Posit Team 2024) were used for all statistical analyses and the R package *tidyverse* v2.0.0 was used for visualization (Wickham et al. 2019). In case other significance levels than *p* < 0.05 were considered, it is mentioned.

Microbial data was examined for sequencing depth using rarefaction curves (Fig. A4) applying the *rarecurve* function implemented in the package *vegan* v2.6.4 (Oksanen et al. 2022). The ASV tables were 100-fold iteratively subsampled to the minimal read number using *rrarefy* implemented in *vegan* to adjust for differences in sequencing depth between samples (Schloss 2024). The α and β-diversity metrics, which were acquired using the functions *diversity* and *vegdist* in *vegan*, respectively, were determined based on the average of 100 subsampled matrices. The effects of the water regime, cropping system, soil layers, and their interaction as well as the effects of the water regime, cropping system, and their interaction in each soil layer separately on β-diversity were analysed by permutational analysis of variance (PERMANOVA) (Anderson 2001) and permutational analysis of multivariate dispersion (PERMDISP) (Anderson 2006) using the *adonis2* and *betadisper* functions in *vegan*. Unconstrained and constrained ordinations were obtained with the principal coordinate analysis (PCoA) using the *cmdscale* function in *vegan* and with canonical analysis of principal coordinates (CAP) (Anderson and Willis 2003) using the *CAPdiscrim* function in the *BiodiversityR* package v2.15.2 (Kindt and Coe 2005), respectively. Depth-separated data was constrained by the water regime and cropping system. To test the individual effects of the water regime, cropping system, and their interaction on taxonomic groups in each soil layer, aggregated read counts across the taxonomic hierarchy of every ASV assigned to the same taxonomic group were tested by PERMANOVA followed by subsequent adjustment for multiple testing using the *qvalue* function in *qvalue* v2.32.0 (Storey and Tibshirani 2003). Z-transformed data were used to visualize differences in relative abundances between the water regime treatments using the *scale* function. Taxonomic trees using iToL v6.8.1 (Letunic and Bork 2011) were created to visualize genera significantly affected by drought based on the taxonomy table using *taxa2dist* in *vegan* and *hclust* in the *ade4* package v1.7–22 (Dray and Dufour 2007).

Linear mixed-effects models were calculated across the entire soil profile (0–0.75 m) (i), for each soil layer separately (0–0.25 m, 0.25–0.5 m, 0.5–0.75 m) (ii), and all soil layers together (iii). The water regime, cropping system, and their interaction were used as fixed effects and field plot as random factor (i + ii) and the water regime, cropping system, soil layer, and their interactions as fixed and field plot as random factor (iii) using the function *lmer* implemented in the package *lmerTest* v3.1.3 (Kuznetsova et al. 2017). Explained variables were (i) mass ^13^C excess of aboveground and belowground biomass, and soil, total net assimilated C, grain C, straw C, fine, coarse, and total root C, rhizodeposition C, relative rhizodeposition C of total belowground C input, root-to-shoot ratio, belowground C to aboveground C ratio and relative C allocation coefficients of grain C, straw C, root C, and rhizodeposition C and (ii + iii) 16S and 18S rRNA gene copy numbers, the ratio of copiotrophs to oligotrophs, α-diversity, GWC, relative rhizodeposition C of total belowground C input, fine, coarse, and total root C, and rhizodeposition C. Differences between means were tested by analysis of variance (ANOVA), subsequently analysed by multiple comparisons of least-square means, and adjusted for multiple testing using Tukey in the packages *emmeans* v1.10.1 and *multcomp* v1.4.25 (Lenth 2024). Normality and homogeneity were visually inspected using QQ and residual plots and in case of non-normality was log- or square-root-transformed. Spearman correlations between rhizodeposition C and fine, coarse, and total root C overall and in each soil layer were performed using the *cor.test* implemented in R.

## Results

### Drought effects on crop growth and soil gravimetric water content

**Fig. 1:**
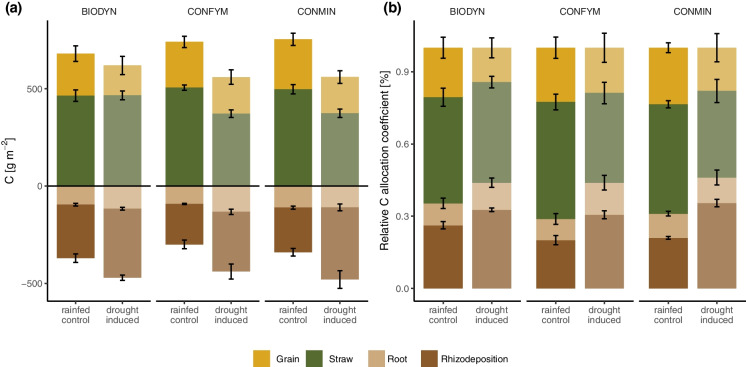
Water regime and cropping system effect on grain, straw, root, and rhizodeposition C at wheat ripening. **a**) Absolute values of grain, straw, root, and rhizodeposition C in the three cropping systems (i.e. BIODYN, biodynamic cropping system; CONFYM, mixed conventional system; and CONMIN mineral fertilized conventional system) and two water regimes. Values of root C and rhizodeposition C over the entire soil profile (0–0.75 m). **b**) Relative C allocation coefficient of grain, straw, root, and rhizodeposition C in the three cropping systems and two water regimes. Means and standard errors are shown

Total average dry matter grain yields in rainfed conditions were 8.0 t ha^−1^ for CONMIN, 7.4 t ha^−1^ for CONFYM, and 6.6 t ha^−1^ for BIODYN, respectively. Below the rainout shelters, ear damage by animals was recorded at grain ripening, as shelters offered protection from predators, resulting in a preference for sheltered plants. One replicate plot (i.e. BIODYN drought-induced) was removed from the grain C analysis since around 20 tillers were missing. In the other sheltered plots, only two ears on average were missing from tillers, minimizing the impact of animal damage. Sheltering significantly reduced grain C by around 25 ± 4% across all cropping systems (Fig. 1a, Table 1). Straw C was reduced by 31 ± 10% in CONFYM and 29 ± 11% in CONMIN but not affected in BIODYN (Fig. 1a, Table 1). The total net assimilated C (i.e. grain C, straw C, root C, and rhizodeposition C) did not significantly differentiate between the control and drought-induced plots (*p* > 0.05; Fig. 1a).

**Table 1:** Effect of water regime, cropping system, and their interactions on grain, straw, total, coarse, fine root C, rhizodeposition C, root-to-shoot ratio (root C/straw C), belowground C to aboveground C ratio (below-to-above; (rhizodeposition C + root C)/(straw C + grain C)) in 0–0.75 m assessed by ANOVA (F-value and p-value) at wheat ripening. Significant values are indicated as bold. Log-transformed data are indicated as^1^

	Grain C	Straw C	Total root C	Coarse root C	Fine root C	Rhizode-position C	Root-to-Shoot^1^	Below-to-above
F-value (*p*-value)								
Water regime (W)	**26.1 (< 0.001)**	**41.7 (< 0.001)**	**5.7** **(0.029**)	0.5 (0.506)	**21.4** **(0.001)**	**46.9** **(< 0.001)**	**17.4** **(0.002)**	**55.7 (< 0.001)**
Cropping system (C)	3.3 (0.063)	0.7 (0.518)	0.2 (0.836)	0.0 (0.979)	0.5 (0.620)	**4.9** **(0.020)**	0.5 (0.613)	1.4 (0.274)
W x C	0.3 (0.695)	**10.9 (0.004)**	2.0 (0.166)	0.6 (0.586)	3.0 (0.104)	1.4 (0.275)	2.7 (0.118)	1.6 (0.236)

**Fig. 2:**
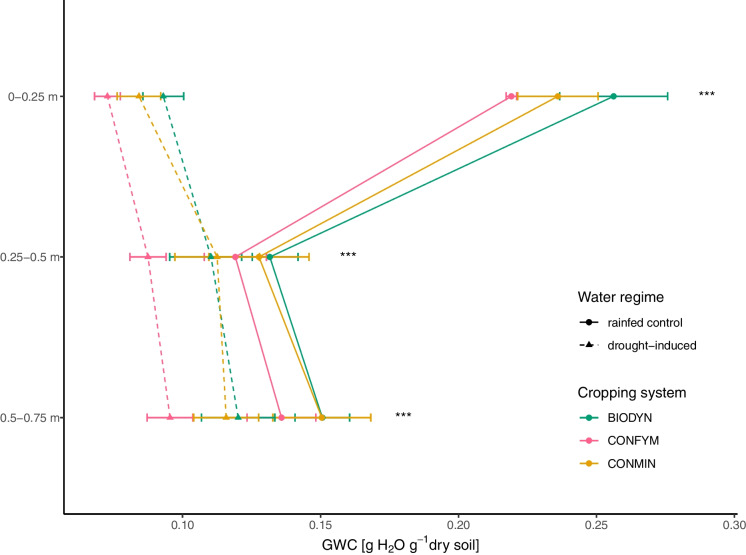
Gravimetric water content (GWC) for each cropping system (i.e. BIODYN, biodynamic cropping system; CONFYM, mixed conventional system; and CONMIN, mineral fertilized conventional system) in drought-induced (dashed lines) and rainfed control (solid lines) plots for all three soil layers at grain ripening (0–0.25 m, 0.25–0.5 m, and 0.5–0.75 m). Asterisks indicate significant (*p* < 0.001, *n* = 12) differences between drought and control plots. Means and standard errors are shown

At grain ripening, there was a significant water reduction in all measured soil layers (Fig. 2), yet the difference between the water regimes was greatest in the topsoil layer (i.e. 65 ± 1% (0–0.25 m) compared to 18 ± 6% (0.25–0.5 m) and 24 ± 3% (0.5–0.75 m)).

### Increased uptake of ^13^C under rainfed conditions

**Fig. 3:**
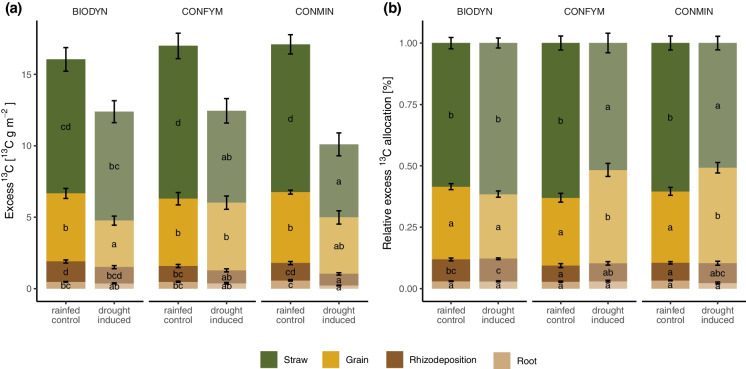
The ^13^C mass excess in straw, grains, roots, and rhizodeposition in the rainfed control and drought-induced plots. **a**) The total ^13^C mass excess in the cropping systems (i.e. BIODYN, biodynamic cropping system; CONFYM, mixed conventional system; and CONMIN, mineral fertilized conventional system), **b**) the percentage of ^13^C mass excess to the total recovered ^13^C in the cropping systems. Means and standard errors are shown. In case of a significant interactive effect of water regime and cropping systems, different letters indicate significant (p < 0.05) differences assessed in the least squares means

The applied ^13^C into the plant-soil system was estimated to be 36.5 g ^13^C m^−2^. Net ^13^C recovery of the plant-soil system was significantly higher in the control with 46% (17.2 ± 0.4 g m^−2^) compared to the drought treatment with 32% (12.0 ± 0.5 g m^−2^, *p* < 0.001; Fig. 3). Significantly more ^13^C excess was found in the straw in the control than in the drought-induced treatments of both conventional systems (e.g. CONMIN and CONFYM; Fig. 3a). In contrast, the lowest total ^13^C excess was found in the BIODYN in the grains under drought (Fig. 3a). While no reduction of the ^13^C excess in root and rhizodeposition was observed under drought in BIODYN and CONFYM, a decrease was found in CONMIN. Relative to the total ^13^C excess recovered, less ^13^C was allocated to shoots and more to grains in the conventional systems under drought, but not in BIODYN. No differences between the control and drought treatments were shown for the relative ^13^C allocation to the root and rhizodeposition in any cropping system (Fig. 3b).

### Increased root and rhizodeposition C under drought

**Fig. 4:**
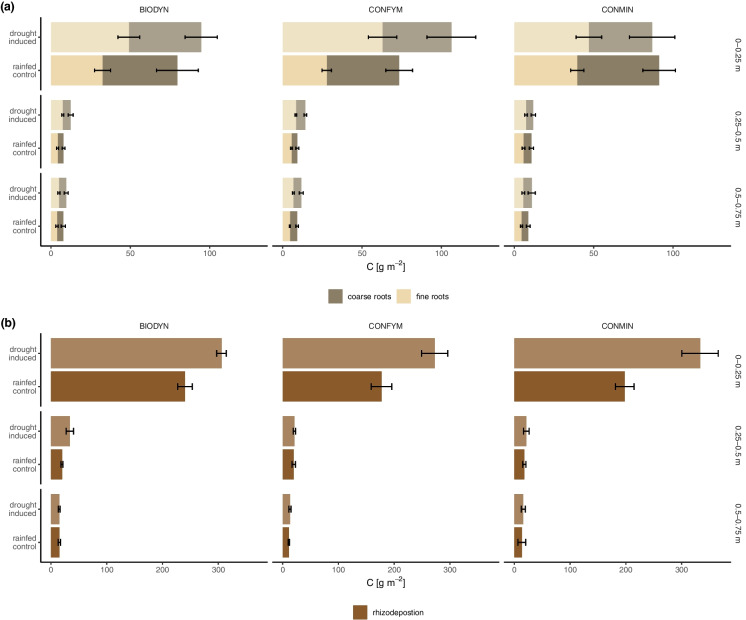
Water regime and cropping system effect (i.e. BIODYN, biodynamic cropping system; CONFYM, mixed conventional system; and CONMIN mineral fertilized conventional system) on fine and coarse root C, and rhizodeposition C at wheat ripening in all three depth layers (i.e. 0–0.25 m, 0.25–0.5 m, and 0.5–0.75 m). a) fine and coarse roots in the three soil layers, b) rhizodeposition in the three soil layers. Means and standard errors are shown

Over the entire soil profile (0–0.75 m), total and fine root C were significantly higher by 20 ± 20% and 56 ± 36%, respectively, in the drought treatment across all cropping systems without any interaction between cropping system and water regime (Fig. 1a, Table 1). When including soil layer as an additional factor in the analysis, total root C increased in response to drought across all cropping systems, and fine root C in CONFYM and BIODYN (Table 2). Analysing soil layers separately, there was significantly higher fine root C under drought in all soil layers across all cropping systems (e.g. 61 ± 42% in 0–0.25 m, 49 ± 21% in 0.25–0.5 m, and 36 ± 10% in 0.5–0.75 m; Fig. 4a, Table A3), while total root C was increased by 38 ± 21% and 26 ± 2%, in 0.25–0.5 m and 0.5–0.75 m, respectively, and coarse root C by 25 ± 27% in 0.25–0.5 m (Fig. 4a, Table A3). No cropping system-water regime interaction was found if soil layers were examined separately.

**Table 2:** Effect of water regime, cropping system, soil layers, and their interactions on total, coarse, and fine root C and rhizodeposition C assessed by ANOVA (F-value and p-value) at wheat ripening. Significant values are indicated as bold. Log- and sqrt-transformed data are indicated as ^1^ and ^2^, respectively

ANOVA	Total root C^1^	Coarse root C^1^	Fine root C^1^	Rhizodeposition C^2^
*F*-value (p-value)				
Water regime (W)	**22.1 (< 0.001)**	1.6 (0.210)	**54.7 (< 0.001)**	**30.4 (< 0.001)**
Cropping system (C)	1.0 (0.399)	0.1 (0.878)	1.6 (0.265)	**4.3 (0.018)**
Soil layer (S)	**857.0 (< 0.001)**	**641.8 (< 0.001)**	**641.6 (< 0.001)**	**1057.8 (< 0.001)**
W x C	2.8 (0.072)	1.5 (0.233)	**3.5 (0.039)**	0.6 (0.574)
W x S	1.2 (0.314)	2.5 (0.091)	0.8 (0.474)	**14.0 (< 0.001)**
C x S	0.5 (0.763)	0.3 (0.876)	0.6 (0.702)	1.3 (0.269)
W x C x S	0.5 (0.755)	0.9 (0.491)	1.1 (0.361)	1.3 (0.286)

Rhizodeposition C was significantly increased in 0–0.25 m, but not in the deeper soil layers, as indicated by a significant interaction between water regime and depth (Fig. 4b, Table 2). This was confirmed by the separate analysis of the soil layers, which showed a significant 48 ± 17% increase in rhizodeposition C at 0–0.25 m in the drought treatment across all cropping systems but no effect in the intermediate and deep soil layers (Fig. 4b, Table A3). No interactive effects of cropping system and water regime on rhizodeposition were found in any of the soil layers separately (Table A3). Rhizodeposition C analysed over the entire soil profile (0–0.75 m) was significantly increased by 45 ± 14% in the drought-induced treatments across all cropping systems (Fig. 1a, Table 1).

### Higher C allocation to belowground under drought

**Fig. 5:**
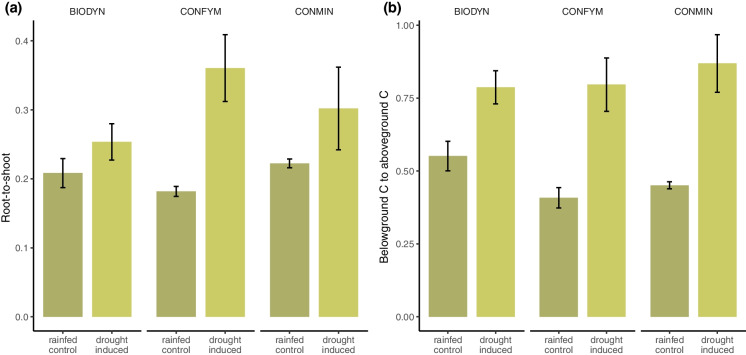
Water regime and cropping system effect (i.e. BIODYN, biodynamic cropping system; CONFYM, mixed conventional system; and CONMIN mineral fertilized conventional system) on root-to-shoot ratio (root C/straw C) and belowground C to aboveground C ratio (rhizodeposition C + root C)/(straw C + grain C) at wheat ripening in across all three depth layers (i.e. 0–0.75 m). a) Root-to-shoot ratio, b) belowground C to aboveground C ratio. Means and standard errors are shown

Drought significantly reduced relative C allocation to grain across all cropping systems (*p* < 0.001; Fig. 1b), while relative more C was allocated to roots under drought (*p* < 0.05; Fig. 1b). More C was relative allocated to rhizodeposition in CONFYM and CONMIN under drought, whereas there was no significant effect of drought in BIODYN (Fig. 1b). Relative to total belowground C, more C was allocated to rhizodeposition than to roots in 0–0.25 m and over the entire soil profile (0–0.75 m) in CONMIN under drought, but not in BIODYN and CONFYM (Fig. A5). The root-to-shoot ratio and belowground C to aboveground C ratio were significantly higher in the drought-induced plots compared to the control in all cropping systems (Fig. 5, Table 1).

Rhizodeposition C significantly correlated with fine root C in 0–0.25 m and 0.25–0.5 m (Fig. A6a & c), while it was not correlated in 0.5–0.75 m (Fig. A6e). Total root C was marginally and significantly correlated with rhizodeposition C in 0–0.25 m and 0.25–0.5 m (Fig. A6b & d), respectively, but not in 0.5–0.75 m (Fig. A6f). No significant correlation was found for coarse root C (*p* > 0.05; data not shown).

### Drought affects microbial composition rather than abundance and diversity

Drought had a marginal effect on the prokaryotic and fungal abundance in the rhizosphere of the topsoil layer (*p* = 0.0565 for prokaryotes, *p* = 0.0591 for fungi), reflected in a slight reduction under drought (Fig. A7a & b). There was an interactive effect for prokaryotic α-diversity of the water regime and cropping system (*p* < 0.05) and water regime, cropping system, and soil layer (*p* < 0.05), although no consistent effect of one cropping system or water regime was observed in these soil layers. Analysing the soil layers separately, we found an interactive effect of the water regime and cropping system in 0.5–0.75 m on the prokaryotic α-diversity (Fig. A7c). In 0.25–0.5 m, a marginally significant increase of fungal α-diversity under drought was observed (Fig. A7d).

An interactive effect of the water regime and depth was found on the oligotroph:copiotroph ratio in the rhizosphere over the entire profile, reflected by larger differences in the topsoil layer compared to the deeper soil layers. The separate analysis of the soil layers confirmed the water regime effect on the oligotroph:copiotroph ratio in 0–0.25 m. No water regime effect was observed in the deeper soil layers.

Prokaryotic and fungal β-diversity in the rhizosphere were significantly affected by water regime and cropping system in 0–25 cm, which was supported by a CAP analysis (Fig. A8), showing a higher variance explained by the cropping system (prokaryotes 32%, fungi 24%) than the water regime (prokaryotes 7%, fungi 18%). While the water regime affected fungal β-diversity in 0.5–0.75 m, no effects were found for prokaryotic β-diversity in this soil layer (Table A4). No interactive effects of the water regime and cropping system were observed in any of the soil layers.

### Drought affected certain prokaryotic and fungal genera

**Fig. 6:**
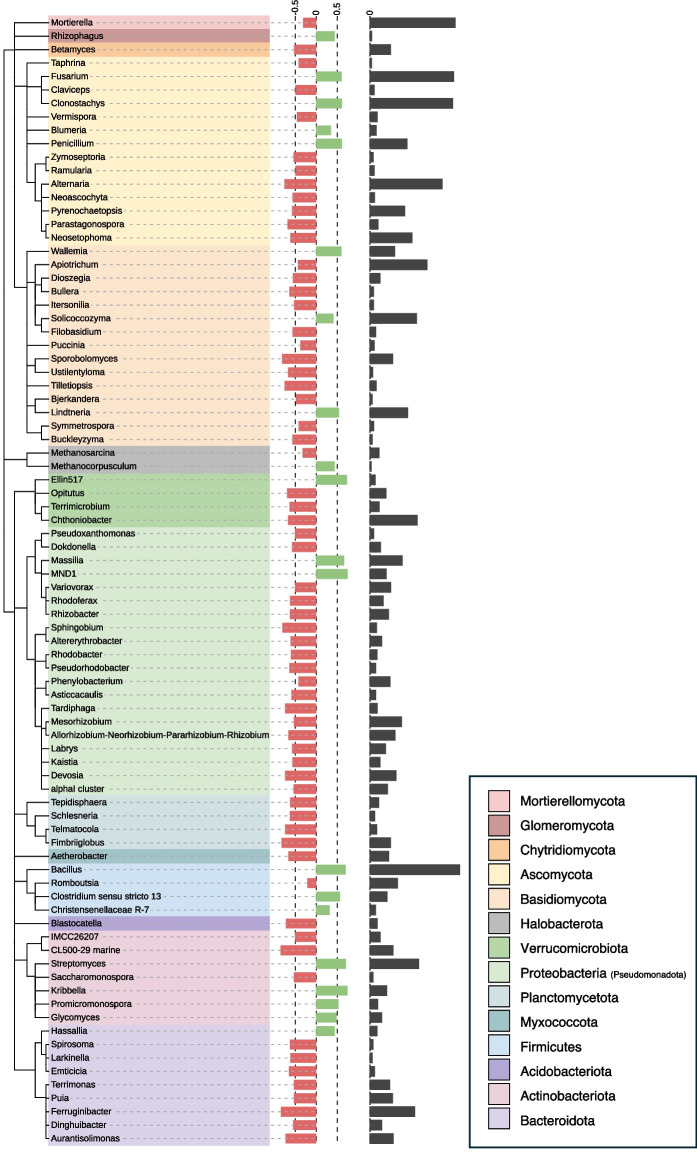
Taxonomic tree of prokaryotic and fungal genera in rhizosphere responding significantly to drought (PERMANOVA, q < 0.05). Color ranges indicate corresponding phyla. Colored bar plots showing the z-transformed relative change in abundance of genera either enriched (green) or depleted (red) under drought. Black bar plots represent the relative square-root transformed mean abundances of genera in the overall community

Around 8% (53 out of 696) of prokaryotic genera and 7% (32 out of 439) of fungal genera were significantly affected (*q* < 0.05) by drought in the wheat rhizosphere across all cropping systems (Fig. 6). On the prokaryotic phylum level, *Bacteroidota*, *Deinococcota*, *Fibrobacterota*, and *Proteobacteria (Pseudomonadota)* were relative increased under drought compared to the control as opposed to *Gemmatimonadota*, *Latescibacterota*, *Synergistota*, *Thermoplasmatota*, which were relative decreased under drought (*q* < 0.05). For fungal phyla, *Basidiobolomycota* and *Rozellomycota* were both relative decreased under drought across all cropping systems compared to the control (*q* < 0.05). No prokaryotic nor fungal phyla or genera showed a significantly different drought response depending on the cropping system.

Drought-responsive prokaryotic genera in the wheat rhizosphere included, but were not limited to *Allorhizobium-Neorhizobium-Pararhizobium-Rhizobium, Altererythrobacter, Asticcacaulis, Devosia, Labrys, Mesorhizobium, MND1, Phenylobacterium, Pseudoxanthomonas, Rhodobacter, Rhodoferax, Sphingobium, Variovorax* (all *Proteobacteria*)*, Emticicia* (*Bacteroidetes*)*, Methanosarcina* (*Euryarchaeota*)*, Glycomyces, Penicillium, Promicromonospora, Saccharomonospora, Streptomyces* (all *Actinobacteriota*)*,* and *Bacillus* (*Firmicutes*). Fungal genera, that were significantly influenced by drought, were for example, *Bjerkandera, Buckleyzyma, Bullera, Puccinia, Solicoccozyma, Tilletiopsis* (all *Basidiomycota*)*, Mortierella* (*Mortierellomycota*)*, Alternaria, Claviceps, Clonostachys, Fusarium, Neoascochyta, Parastagonospora, Pyrenochaetopsis, Taphrina, Zymoseptoria* (all *Ascomycota*)*.*

## Discussion

### Drought effect on soil water and aboveground biomass C

Drought conditions were successfully established in 0–0.25 m (0.08 vs 0.24 g H_2_O g^−1^ soil; Fig. 2). Previous studies described a soil water content of around 10% as a severe drought (Hueso et al. 2011; Liu et al. 2022; Jaeger et al. 2023), thus severe drought conditions were induced in the topsoil layer. Although there was a significant difference in soil water content in the soil layers below 25 cm, the reduction of soil water was only around 20% lower in the drought-induced plots as compared to the controls.

An aboveground biomass C of around 725 g m^−2^ under rainfed conditions was found, which is similar to aboveground biomass C found in other studies for wheat (Jensen 1993; Hirte et al. 2018a). Drought-induced plants in conventional systems had around 30% lower straw C than the control (Fig. 1a), while no difference was found in the biodynamic system. One reason might be that the applied plant growth regulators in conventional systems changed the phytohormonal profile under drought and led to differential plant growth (Dwivedi et al. 2019). Another reason might be that winter wheat variety Wiwa has been organically bred such that the plants might be better adapted for the organic systems and consequently deal better with stress conditions (Murphy et al. 2007). Grain C was reduced by sheltering in all cropping systems (Fig. 1), which was strongly driven by the higher damage of wheat by rodents below the shelters. Since wheat ears were eaten by animals only at the very end of the season, it likely did not influence C assimilation before ripening.

### Drought effect on belowground biomass C

Root C under rainfed conditions were on average 99 g C m^−2^. Although the measured root C inputs were slightly higher than reported in a previous study in the same field trial, they are still within the reported range of around 50–240 g m^−2^ (Swinnen 1994; Hirte et al. 2018a, 2021; Van de Broek et al. 2020; Heinemann et al. 2023). In general, the root C inputs depend on the genotype (Van de Broek et al. 2020), field site (Bolinder et al. 1997; Hirte et al. 2018b), season (Chirinda et al. 2012), and sampling timepoint. A root-to-shoot ratio in the range of 0.09–0.80 for wheat has been reported in the literature (Hirte et al. 2018b; Van de Broek et al. 2020; Heinemann et al. 2023), which is in line with our results (Fig. 5).

We found significantly higher absolute root C under drought compared to the control across the entire soil profile for all cropping systems (Fig. 1a, Table 1), as well as at all depth layers (Fig. 4a, Table 2). In addition, we found an increased root-to-shoot ratio (Fig. 5, Table 1) and root C of total C (Fig. 1b) under drought. Hence, part of our first hypothesis, that allocation of C to roots will increase under drought, was corroborated. Other studies found a decrease in total root biomass under drought (Ehdaie et al. 2012; Zhou et al. 2018; Wang et al. 2021), but higher C allocation to roots relative to total plant C has been reported under drought (Fig. 1b) (Palta and Gregory 1997; Ehdaie et al. 2012; Zhou et al. 2018). The effects of drought on C allocation to roots have been shown to depend on drought length and severity (Meharg and Killham 1990; Liang et al. 2017; Zhou et al. 2018). The increase in root C was driven by the increase in fine root C in all measured soil depth layers (Fig. 4a, Table A3). It has been reported that root systems proportionally grow thinner roots under drought conditions (Xiao et al. 2020), likely to improve nutrient and water acquisition (Jackson et al. 1997; Comas et al. 2012). An increased root-to-shoot ratio under drought (Table 1, Fig. 5), which has been previously described (Zhou et al. 2018; Bacher et al. 2022), has been shown to improve water uptake (Bacher et al. 2022).

The average amount of rhizodeposition C of 237 g m^−2^ reported in this field trial under rainfed conditions was higher than previously observed values of 60–170 g m^−2^ (Swinnen 1994; Swinnen et al. 1994a; Hirte et al. 2018a; Sun et al. 2018; Van de Broek et al. 2020). However, many studies describe rhizodeposition in proportion to total belowground C rather than the absolute amounts. Relative rhizodeposition to total belowground C of around 71% was found in our study under rainfed conditions, which is within the range of 45–74% reported (Swinnen 1994; Hirte et al. 2018a; Sun et al. 2018; Van de Broek et al. 2020). It is important to mention that rhizodeposition C in our current study included C in fine roots and root fragments smaller than 0.5 mm, which is a common practice when measuring rhizodeposition in the field (Swinnen et al. 1994a, b; Wichern et al. 2008; Hirte et al. 2018a, b).

There was an increase in absolute C rhizodeposition by 45% under drought compared to the control, primarily in the topsoil layer (Fig. 1a and 3b) where the strongest water reduction was found (Fig. 2). The lack of significant differences in rhizodeposition between the control and drought-induced plots in the intermediate and deep soil layers might be attributed to the smaller observed difference in soil moisture in these deeper layers (Fig. 2). Moreover, relative C rhizodeposition of total C increased under drought (Fig. 1b). This is in line with our first hypothesis that belowground C input will increase under drought. It has been previously found that the rhizodeposition per gram plant biomass of dicots decreased or were unaffected by water limitation, whereas monocots’ rhizodeposition (i.e. wheat) were either unaffected or increased (Preece and Peñuelas 2016). These findings are not supported by studies performed in a greenhouse, which found a decrease in wheat rhizodeposition by plant biomass under drought (Palta and Gregory 1997; Canarini and Dijkstra 2015; Bakhshandeh et al. 2019). However, the drought response of rhizodeposition can differ between field and greenhouse (Meharg and Killham 1990), wheat varieties (Van de Broek et al. 2020), drought severity (Preece and Peñuelas 2016), and sampling timepoints (Swinnen et al. 1994b).

The increase in rhizodeposition C accompanied by the increase of fine root C under drought suggests that rhizodeposition C is driven by fine root C. On the one hand, fine root fragments might have passed the 0.5 mm sieve and therefore contributed to the calculated rhizodeposition (Shamoot et al. 1968; Lynch and Whipps 1990; Oburger and Jones 2018). On the other hand, it was also shown that exudation by gram root increases with decreasing root diameter (Akatsuki and Makita 2020), suggesting that the increase of fine root C under drought would increase rhizodeposition. Supporting the possibility that fine roots contributed to rhizodeposition or increased exudation by finer roots, we found significant correlations between fine root C and rhizodeposition C (Fig. A6), which decreased from the topsoil to the intermediate layer and was insignificant in the deepest soil layer.

The increase in C rhizodeposition under drought stress could indicate a tolerance (i.e. an upregulation of root activity to forage for nutrients and water) or a susceptibility to drought (i.e. root damage and mortality) (Preece and Peñuelas 2016). The accumulation of net rhizodeposition could be also caused by a decreased consumption of less active microbes under drought (Ren et al. 2018). Moreover, previous studies showed that while plants reduced aboveground growth under drought, they maintained their photosynthesis and transported the surplus C belowground (Muller et al. 2011; Prescott et al. 2020). Root and rhizodeposition C are considered to be important for long-term C storage in soils (Poeplau et al. 2021; Villarino et al. 2021), but further studies are required to assess the fate of C after drought. For example, Canarini and Dijkstra (2015) found no effect of drying and wetting cycles on soil C stabilization potential.

### Cropping system effect on the drought response of roots and rhizodeposition

Total root C did not show a cropping system-dependent drought response. However, we could detect an interactive effect of drought and cropping system on the fine root C biomass analysed across all depths (Table 2). While the fine root C in BIODYN and CONFYM showed a significant increase under drought, the fine root C in CONMIN was not significantly higher under drought than in the control. Thus, our second hypothesis, that root C during drought will increase more under organic fertilization compared to cropping systems that are exclusively mineral fertilized, was partly corroborated. Under rainfed conditions, it has been shown that root biomass increased in organic farming (Chirinda et al. 2012; Hu et al. 2018; Hirte et al. 2021), which was attributed to excessive root growth triggered by weeds (Depuydt 2014), foraging for soil resources under decreased mineral nitrogen fertilization, and increased soil organic carbon in organic systems possibly increasing soil aeration and stimulating root growth (Hirte et al. 2021). Under drought, increased soil aeration and weeds might play a minor role, whereas fertilization can affect the root drought response (Wang et al. 2021). Since BIODYN and CONFYM are fully and partly organically fertilized systems, respectively, roots might have to spread more to search for nutrients in organically fertilized soils, where nutrients may be less available compared to CONMIN, especially during drought. However, CONFYM received mainly mineral fertilizers during this wheat vegetation period and received only 20 kg mineral nitrogen per hectare less than CONMIN (130 vs 150 kg ha^−1^) (Table A1), but the long-term effects of increased soil organic C in CONFYM (Krause et al. 2022) might still increase aggregate stability, decrease bulk density, enhance porosity, and influence soil nutrient availability (Bashir et al. 2021), potentially leading to increased root growth. However, more studies are required to assess the effect of farmyard manure application on fine root C under drought conditions to gain a more mechanistic understanding.

No difference in the drought response between the cropping systems on total rhizodeposition C could be detected, i.e. all cropping systems showed an increase of rhizodeposition C under drought (Fig. 1 & 4b), which is contrary to our second hypothesis that rhizodeposition will vary depending on the agricultural management during drought. Thus, while no increase in fine root C in CONMIN was found under drought, the rhizodeposition increased in 0–25 m (Fig. 4). The reasons for this increase of rhizodeposition without an increase in fine root C in CONMIN are unclear. It could be that root mortality in CONMIN under drought was higher, leading to higher rhizodeposition but no higher fine root C (Fig. 4, Table 2) (Preece and Peñuelas 2016). As mentioned above, a part of the calculated rhizodeposition might also consist of fine root fragments, which have passed the 0.5 mm sieve (Shamoot et al. 1968; Lynch and Whipps 1990; Oburger and Jones 2018). This could mean that wheat in CONMIN might have a similar increase in fine root C, but due to the potential increased root mortality under drought, they would be measured here as rhizodeposition. It is also possible that the quality of fine roots differs between the cropping systems, resulting in variable degradation rates. On the other hand, higher belowground C allocation to rhizodeposition under drought in CONMIN could indicate higher root exudation activity by root biomass for lubrication or nutrient foraging, possibly suggesting a different foraging strategy compared to BIODYN and CONFYM.

### Drought-affected microbial response to rhizodeposition

Changes in rhizodeposition under drought can influence soil microbes (Preece and Peñuelas 2016; Williams and de Vries 2020). Although we measured an increase of rhizodeposition C under drought, no significant effect of drought was found on microbial abundance in the rhizosphere, assessed as gene copy number, and α-diversity (Fig. A7). However, more copiotrophic prokaryotes were found under drought, compared to the control, in the topsoil layer (Fig. A7e), which could indicate a decrease in the prokaryotic cell abundance under drought because copiotrophs usually carry a higher number of rRNA gene copies per cell. Since microbes are less active under water-limited conditions as indicated previously in the same field trial (Kost et al. 2024), they might have not been able to efficiently use the rhizodeposition, which led to an accumulation of deposited C under drought and a lack of increase in microbial abundance. The increased ratio of copiotrophic prokaryotes compared to oligotrophic under drought in the rhizosphere could be caused by the increased rhizodeposition under drought favouring copiotrophic lifestyles (López et al. 2023).

A strong effect of drought was found on fungal and prokaryotic community composition in the rhizosphere of the topsoil layer (Table A4, Fig. A8). It has been shown that microbial communities in closer plant association are more strongly affected by drought (Santos-Medellín et al. 2017), which can be attributed to both direct drought effects (e.g. low nutrient availability, higher osmotic potential) and indirect effects of drought-stressed plants such as altered rhizodeposition quantity and quality. Moreover, although the soil water content is higher in the rhizosphere than the bulk soil during drought, possibly due to mucilage exudation (Carminati et al. 2010), microbes may compete with plants for water, consequently increasing drought stress for rhizosphere microbes.

Several rhizosphere microbes (e.g. *Streptomyces, Bacillus, Massilia, Glycomyces, Promicromonospora, MND1* (all bacteria), *Solicoccozyma, Penicillium, Rhizophagus,* and *Clonostachys* (all fungi)) containing potential PGP species were significantly enriched in the topsoil layer under drought (Fig. 6). Some taxa of these genera can produce siderophores, auxin, and aminocyclopropane-1-carboxylate (ACC) deaminase, improve plant phosphorus uptake, fix nitrogen, and have biocontrol capabilities (Aroca et al. 2008; Srinivasan et al. 2020; Sun et al. 2020; Li et al. 2023; Carvajal et al. 2024). Siderophores can help increase plant iron uptake and suppress plant pathogens (Saha et al. 2013). Auxin is involved in the growth of lateral roots and root hairs (Saini et al. 2013), which can facilitate water uptake (Ahmed et al. 2016; Cai and Ahmed 2022). The ACC deaminase can reduce the plant ethylene concentration, which can decrease plant growth under stress (Glick et al. 1998). *Blumeria* and *Fusarium* were enriched under drought in the rhizosphere (Fig. 6). These genera contain plant pathogenic species, known to infest wheat (Zhang et al. 2005; Dweba et al. 2017). Yet, no symptoms of these diseases were detected (data not shown). Symptoms of spot blotch on ears and leaves, caused by *Cochliobolus sativus* (Gupta et al. 2018), were more often detected on sheltered wheat but no relative increase under drought of this genus was found (Fig. 6).

Some taxa including PGP species decreased under drought in the rhizosphere, which have been reported to be involved in the mechanisms mentioned above, yet most of them share the trait of nitrogen fixation in common (i.e. *Tardiphaga, Allorhizobium-Neorhizobium-Pararhizobium-Rhizobium, Rhodoferax, Rhodobacter, Labrys, Mesorhizobium, Saccharomonospora, Pseudoxanthomonas, Phenylobacterium,* and *Methanosarcina*; Fig. 6) (De Lajudie et al. 1998; Kuykendall et al. 2015; Safronova et al. 2015; Nafis et al. 2019; Li et al. 2023). This may seem contradictory to the found increased rhizodeposition during drought since exudates can support nitrogen-fixing prokaryotes (Beck and Gilmour 1983). However, nitrogen fixation is an energy-costly process and might be limited under water limitation (Sun et al. 2021). It was suggested that microbial activity might be reduced under drought in this field experiment (Kost et al. 2024), indicating that microbial processes such as nitrogen fixation might have been decreased under drought. Potential PGP taxa (*Variovorax, Sphingobium, Asticcacaulis, Emticicia,* and *Devosia*), which are commonly found associated with water or under water-enriched conditions (Ngo et al. 2017; Xu et al. 2023), were reduced under drought (Fig. 6). *Buckleyzyma, Bjerkandera, Mortierella, Bullera,* and *Tilletiopsis*, which mostly are involved in biocontrol activity but also exhibit above mentioned PGP abilities, were decreased under drought (Fig. 6). Lastly, many potential fungal plant pathogens were decreased under drought (i.e. *Alternaria, Parastagonospora, Ustilentyloma, Pyrenochaetopsis, Neoascochyta, Zymospeotroira, Itersonilia, Ramularia, Claviceps, Taphrina,* and *Puccinia;* Fig. 6) (Bauer et al. 2001; Rodrigues and Fonseca 2003; Mercado Vergnes et al. 2006; McGovern et al. 2006; Bolton et al. 2008; Menzies and Turkington 2015; Palma-Guerrero et al. 2016; Da Silva et al. 2019; Golzar et al. 2019; Kariyawasam et al. 2023). Although it is unclear why potential plant pathogens decreased under drought, it could be that the required humidity for infections and reproduction is lacking (Aung et al. 2018) or root exudates, as part of increased rhizodeposition under drought, can have antimicrobial effects (Bais et al. 2006). No effects of drought on specific genera were found in the deeper soil layers.

Overall, the data support our third hypothesis that bacterial and fungal taxa in the rhizosphere known to increase plant drought tolerance through phytohormone production, stress hormone modulation, nutrient mobilization, or biocontrol activity can increase under drought, potentially being attracted by specific root exudates released under drought (Williams and de Vries 2020). However, these taxa might also be enriched due to direct drought effects. More targeted studies are required to assess a direct connection between rhizodeposition and microbes. Stable isotope probing could give insights into the flow of C to microbes and even metabolites (Nannipieri et al. 2020), but the continuous labelling of ^13^C-CO_2_ needed to reach sufficient labelling of microbial DNA (Bernard et al. 2007; Haichar et al. 2008) is not feasible in the field.

## Conclusion

Under severe drought, wheat crops invest more net assimilated C belowground via fine root growth and rhizodeposition, potentially affecting nutrient and water acquisition of plants, and C storage under future climate conditions. Fine root C showed a cropping system-specific response to drought, suggesting that differently managed systems have contrasting drought responses and underscoring the critical role of agricultural management in shaping the overall impact of climate change on the plant-soil system. Further research is required to assess the effects of agricultural management, such as farmyard manure application, on fine root C under drought and rewetting to improve our knowledge about the underlying processes. We further found that several plant-growth-promoting microbes potentially involved in improving plant drought tolerance were enriched under drought, with the notable exception of microbes involved in nitrogen fixation showing the opposite response. It remains to be determined, however, if these enriched taxa confer a functional benefit to plants.

## Supplementary Information

Below is the link to the electronic supplementary material.

Supplementary file1 (DOCX 25.9 MB)

## Data Availability

Raw sequence data were deposited in the European Nucleotide Archive under the accession number PRJEB80808. Other data are available from the corresponding author on reasonable request.
