# Supplementary material for: Drought increases root and rhizodeposition carbon inputs into soils
Source: Plant Soil. 2026 Jan 12;519(1):103–27. doi: 10.1007/s11104-025-08021-1 (PMC12948865; doi:10.1007/s11104-025-08021-1)
Supplement: Supplementary file 1 — Supplementary file1 (DOCX 25.9 MB) [file 11104_2025_8021_MOESM1_ESM.docx]

# Supplementary Information

## Article title

Drought increases root and rhizodeposition carbon inputs into soil

## Journal name

Plant and Soil

## Authors

Elena Kost^1^, Dominika Kundel^2^, Matti Barthel^1^, Rafaela Feola Conz^1^, Roland Anton Werner^1^, Shiva Ghiasi^3^, Tabata Aline Bublitz^4^, Paul Mäder^2^, Hans-Martin Krause^2^, Johan Six^1^, Martin Hartmann^1^ and Jochen Mayer^3^

## Affiliations

^1^Institute of Agricultural Sciences, Department of Environmental Systems Science, ETH Zürich, Zurich, Switzerland; ^2^Departement of Soil Science, Research Institute of Organic Agriculture, Frick, Switzerland; ^3^Nutrient Flows, Department of Agroecology and Environment, Agroscope, Zurich, Switzerland; ^4^Department of Soil Biology and Plant Nutrition, Faculty of Organic Agricultural Sciences, University of Kassel, Kassel, Germany

## Corresponding Author

Elena Kost, [elena.kost@usys.ethz.ch](mailto:elena.kost@usys.ethz.ch), Universitaetstrasse 2, 8092 Zurich, CH

The following Supporting Information is available for this manuscript:

**Table A1:** Detailed timeline of agricultural interventions.

**Table A2:** Overview of PCR and qPCR conditions.

**Table A3:** Statistical results of total, coarse, and fine root C and rhizodeposition C by each depth.

**Table A4:** Statistical results of prokaryotic and fungal β-diversity.

**Fig. A1:** Pictures of wheat labelling.

**Fig. A2:** Pictures of microplot sampling.

**Fig. A3:** Visual protocol of root and rhizosphere protocol.

**Fig. A4:** Rarefaction curves for fungal and prokaryotic samples.

**Fig. A5:** Results of relative rhizodeposition to total below-ground carbon.

**Fig. A6:** Results of correlation of rhizodeposition and root C.

**Fig. A7:** Results of microbial abundance, α-diversity, and oligotroph:copiotroph ratio.

**Fig. A8:** Results of prokaryotic and fungal community analysis.

**Table A1** Detailed timeline of the agricultural interventions in the rainout-sheltering experiment in the DOK long-term field trial. Samples were collected at ripening. Labeling was performed 11 times from stem elongation to before ripening (in bold).

| 2021-10-07 | Composted manure application in BIODYN (10 t ha^-1^; 63 kg N ha ^-1^, 21 kg P ha^-1^, 76 kg K ha^-1^)  Stacked manure application in CONFYM (12 t ha^-1^; 83 kg N ha^-1^, 15 kg P ha^-1^, 90 kg K ha^-1^)  Mineral fertilizer application in CONMIN (29 kg P ha ^-1^, 75 kg K ha^-1^) |
| --- | --- |
| 2021-10-15 | Primary tillage with disk harrow in all systems |
| 2021-10-19 | Seedbed preparation with rotary harrow and sowing of winter wheat variety Wiwa in all systems (dressed seeds in conventionally managed systems) |
| 2021-11-10 | Installation microplots |
| 2021-11-15 – 11-17 | Installation of rainout-shelter with foil |
| 2021-12-01 – 12-15 | Installation TDR, TOMST, PAR sensors  Installation of rain gutters and IBC tanks |
| 2021-12-22 | Connection lower side of rain gutters with the pipe system  Homogenization plants in microplot 35 ± 2 |
| 2022-01-06 | Installation of respiration chambers |
| 2022-01-20 | Connection higher side of rain gutters to IBC tanks and pipe system |
| 2022-01-28 – 02-02 | Irrigation sheltered plots with 30 mm |
| 2022-02-18 | Irrigation sheltered plots with 15 mm |
| 2022-03-01 | Irrigation sheltered plots with 10 mm |
| 2022-03-10 | Mechanical weeding by hand in the BIODYN at Zadok stage 23 |
| 2022-03-15 | Biodynamic preparations in BIODYN (Horn manure 96 g ha^.1^) at Zadok stage 23 |
| 2022-03-16 | First slurry application in BIODYN (2.5 l ha^-1^) at Zadok stage 23  First mineral N application in conventionally managed systems (60 kg N ha^-1^, 14 kg P ha^.1^, 14 kg K ha^.1^ in CONFYM and 70 kg N ha^-1^ in CONMIN) at Zadok stage 23 |
| 2022-03-28 | Application of herbicide (Othello ® Star; Bayer AG, Leverkusen, Deutschland) and plant growth regulators (CCC 720 ®; Bayer AG) in conventionally managed systems (1 and 0.5 l ha^-1^, respectively) at Zadok stage 30 |
| 2022-03-31 | Irrigation sheltered plots with 2 mm  Installation of HOBO sensors |
| **2022-04-05** | CO_2_, N_2_O, CH_4_ measurement  **Labelling each labelling chamber with 60 mL ^13^C-CO_2_ at Zadok stage 30** |
| 2022-04-11 | Application of plant-growth regulator (Moddus; Syngenta, Basel, Switzerland) in  conventionally managed systems (0.6 l ha^-1^) |
| 2022-04-12 | CO_2_, N_2_O, CH_4_ measurement  **Labelling each labelling chamber with 180 mL ^13^C-CO_2_ at Zadok stage 31** |
| 2022-04-13 | Second slurry application in BIODYN (4 l ha^-1^) at Zadok stage 32  Second mineral N application in conventionally managed systems (30 kg N ha^-1^ in  CONFYM and 40 kg N ha^-1^ in CONMIN) |
| **2022-04-19** | CO_2_, N_2_O, CH_4_ measurement  **Labelling each labelling chamber with 420 mL ^13^C-CO_2_ at Zadok stage 33** |
| 2022-04-21 | Mechanical weeding by hand in BIODYN at Zadok stage 33 |
| **2022-04-26** | CO_2_, N_2_O, CH_4_ measurement  **Labelling each labelling chamber with 420 mL ^13^C-CO_2_ at Zadok stage 34** |
| **2022-05-03** | CO_2_, N_2_O, CH_4_ measurement  **Labelling each labelling chamber with 660 mL ^13^C-CO_2_ at Zadok stage 36** |
| 2022-05-05 | Biodynamic preparations in BIODYN (Horn silica 8 g ha^-1^) |
| **2022-05-10** | CO_2_, N_2_O, CH_4_ measurement  **Labelling each labelling chamber with 660 mL ^13^C-CO_2_ at Zadok stage 38** |
| **2022-05-17** | CO_2_, N_2_O, CH_4_ measurement  **Labelling each labelling chamber with 660 mL ^13^C-CO_2_ at Zadok stage 39**  Third mineral N application in conventionally managed systems (40 kg N ha^-1^ in  CONFYM and 40 kg N ha^-1^ in CONMIN) at Zadok stage 39 |
| 2022-05-18 | Application of fungicide (Aviator® Xproin; Bayer AG) conventionally managed systems  (1.25 l ha^-1^) at Zadok stage 39 |
| **2022-05-24** | CO_2_, N_2_O, CH_4_ measurement  **Labelling each labelling chamber with 720 mL ^13^C-CO_2_ at Zadok stage 61** |
| 2022-05-28 | Biodynamic preparations in BIODYN (Horn silica 8 g ha^-1^) at Zadok stage 61 |
| **2022-05-31** | CO_2_, N_2_O, CH_4_ measurement  **Labelling each labelling chamber with 660 mL ^13^C-CO_2_ at Zadok stage 69** |
| **2022-06-10** | CO_2_, N_2_O, CH_4_ measurement  **Labelling each labelling chamber with 660 mL ^13^C-CO_2_ at. growtZadok stage 73** |
| **2022-06-14** | CO_2_, N_2_O, CH_4_ measurement  **Labelling each labelling chamber with 480 mL ^13^C-CO_2_ at Zadok stage 75** |
| 2022-06-17 – 06-17 | Distribution of net against bird damage |
| 2022-06-21 | CO_2_, N_2_O, CH_4_ measurement |
| 2022-06-28 | CO_2_, N_2_O, CH_4_ measurement |
| 2022-06-29 – 06-30 | Sampling at ripening (first day rainfed control, second day drought plots) at Zadok stage 93 |
| 2022-07-05 | CO_2_, N_2_O, CH_4_ measurement |
| 2022-07-06 – 07-07 | Removing rainout-shelters and HOBO, TDR, PAR sensors |

**Table A2** Overview of PCR and qPCR conditions performed in this study including primer sequences, cycling conditions, PCR reagents, and references.

| Analysis | Primer | Cycling | | | | Mastermix | Ref |
| --- | --- | --- | --- | --- | --- | --- | --- |
| PCR 16S in rhizosphere | 341F (5’-CCTAYGGGDBGCWSCAG-3’)  806R (5’-GGACTACNVGGGTHTCTAAT-3’) | 1 | 2 min | 95 °C |  | 1x GoTaq® G2 Hot start Master mix  0.5 mM MgCl_2_  0.4 µM primer F  0.4 µM primer R  40 ng DNA | [1] |
|  |  | 2 | 40 sec | 95 °C |  |  |  |
|  |  | 3 | 40 sec | 58 °C |  |  |  |
|  |  | 4 | 1 min | 72 °C | 30 × |  |  |
|  |  | 5 | 10 min | 72 °C |  |  |  |
| PCR ITS in rhizosphere | 5.85-Fung (5'-AACTTTYRRCAAYGGATCWCT-3′)  ITS4-Fung (5'-AGCCTCCGCTTATTGATATGCTTAART-3′) | 1 | 2 min | 95 °C |  | 1x GoTaq® G2 Hot start Master mix  1 mM MgCl_2_  0.4 µM primer F  0.4 µM primer R  40 ng DNA | [2] |
|  |  | 2 | 40 sec | 95 °C |  |  |  |
|  |  | 3 | 40 sec | 58 °C |  |  |  |
|  |  | 4 | 1 min | 72 °C | 35 × |  |  |
|  |  | 5 | 10 min | 72 °C |  |  |  |
| qPCR inhibition test | SP6 (5'-ATTTAGGTGACACTATAG-3')  T7 (5'-TAATACGACTCACTATAGGG-3') | 1 | 3 min | 98 °C |  | 1x SsoAdvanced universal SYBR® Green supermix  0.75 µM primer F  0.75 µM primer R  20 ng DNA  10^4^ copy number pGEM-T plasmid | [3] |
|  |  | 2 | 15 sec | 95 °C |  |  |  |
|  |  | 3 | 30 sec | 48 °C |  |  |  |
|  |  | 4 | 30 sec | 72 °C |  |  |  |
|  |  | 5 | 15 sec | 80 °C | 30 × |  |  |
|  |  | 6 | 5 sec | 65-95 °C | 0.3 °C |  |  |
| qPCR 16S in rhizosphere | 515Y-F (modified) (5'-GTGYCAGCMGCCGCGGTAA-3')  806R (original) (5'-GGACTACHVGGGTWTCTAAT-3') | 1 | 3 min | 98 °C |  | 1x SsoAdvanced universal SYBR® Green supermix  0.75 µM primer F  0.75 µM primer R  20 ng DNA | [1,4] |
|  |  | 2 | 15 sec | 95 °C |  |  |  |
|  |  | 3 | 30 sec | 52 °C |  |  |  |
|  |  | 4 | 30 sec | 72 °C |  |  |  |
|  |  | 5 | 15 sec | 80 °C | 35 × |  |  |
|  |  | 6 | 5 sec | 75-95 °C | 0.5 °C |  |  |
| qPCR 18S in rhizosphere | FR1 (5'-ANCCATTCAATCGGTANT-3')  FF390 (5'-CGATAACGAACGAGACC-3') | 1 | 3 min | 98 °C |  |  | [5] |
|  |  | 2 | 20 sec | 95 °C |  |  |  |
|  |  | 3 | 30 sec | 51 °C |  |  |  |
|  |  | 4 | 30 sec | 72 °C |  |  |  |
|  |  | 5 | 15 sec | 80 °C | 40 × |  |  |
|  |  | 6 | 5 sec | 65-95 °C | 0.3 °C |  |  |

References

[1] B. Frey, T. Rime, M. Phillips, B. Stierli, I. Hajdas, F. Widmer, M. Hartmann, Microbial diversity in European alpine permafrost and active layers, FEMS Microbiology Ecology 92 (2016) 1–17. https://doi.org/10.1093/femsec/fiw018.

[2] D.L. Taylor, W.A. Walters, N.J. Lennon, J. Bochicchio, A. Krohn, J.G. Caporaso, T. Pennanen, Accurate estimation of fungal diversity and abundance through improved lineage-specific primers optimized for Illumina amplicon sequencing, Applied and Environmental Microbiology 82 (2016) 7217–7226. https://doi.org/10.1128/AEM.02576-16.

[3] A.C.H. Jaeger, M. Hartmann, J. Six, E.F. Solly, Contrasting sensitivity of soil bacterial and fungal community composition to one year of water limitation in Scots pine mesocosms, FEMS Microbiology Ecology (2023). https://doi.org/https://doi.org/10.1093/femsec/fiad051.

[4] A.E. Parada, D.M. Needham, J.A. Fuhrman, Every base matters: Assessing small subunit rRNA primers for marine microbiomes with mock communities, time series and global field samples, Environmental Microbiology 18 (2016) 1403–1414. https://doi.org/10.1111/1462-2920.13023.

[5] E.J. Vainio, J. Hantula, Direct analysis of wood-inhabiting fungi using denaturing gradient gel electrophoresis of amplified ribosomal DNA, Mycological Research 104 (2000) 927–936. https://doi.org/10.1017/S0953756200002471.

**Table A3** *Effect of water regime, cropping system, and their interactions on total, coarse, fine root C and rhizodeposition C in 0-0.25 m, 0.25-0.5 m, and 0.5-0.75 m, assessed by ANOVA (F-value and p-value) at wheat ripening. Values p < 0.05, p < 0.01, and p < 0.001 are indicated as *, **, and ***, respectively.*

| Total root C | 0-0.25 m | | 0.25-0.5 m | | 0.5-0.75 m | |
| --- | --- | --- | --- | --- | --- | --- |
|  | F-value | p-value | F-value | p-value | F-value | p-value |
| Water regime (W) | 3.06 | 0.0973 | 21.11 | 0.0013 ** | 12.27 | 0.0067 ** |
| Cropping system (C) | 0.04 | 0.9630 | 1.20 | 0.3461 | 0.59 | 0.5745 |
| W x C | 1.67 | 0.2125 | 2.41 | 0.1464 | 0.14 | 0.8732 |
|  |  |  |  |  |  |  |
| Coarse root C | 0-0.25 m | | 0.25-0.5 m | | 0.5-0.75 m | |
|  | F-value | p-value | F-value | p-value | F-value | p-value |
| Water regime (W) | 1.08 | 0.313 | 5.84 | 0.0388 * | 2.87 | 0.1246 |
| Cropping system (C) | 0.05 | 0.951 | 0.40 | 0.6796 | 0.16 | 0.8568 |
| W x C | 0.43 | 0.658 | 3.33 | 0.0828 | 0.24 | 0.7930 |
|  |  |  |  |  |  |  |
|  | 0-0.25 m | | 0.25-0.5 m | | 0.5-0.75 m | |
| Fine root C | F-value | p-value | F-value | p-value | F-value | p-value |
| Water regime (W) | 14.56 | 0.0041 ** | 26.83 | 0.0007 *** | 13.31 | 0.0053 ** |
| Cropping system (C) | 0.26 | 0.7789 | 1.45 | 0.2852 | 1.35 | 0.3065 |
| W x C | 2.53 | 0.1347 | 0.94 | 0.4247 | 0.71 | 0.5159 |
|  |  |  |  |  |  |  |
|  | 0-0.25 m | | 0.25-0.5 m | | 0.5-0.75 m | |
| Rhizodeposition C | F-value | p-value | F-value | p-value | F-value | p-value |
| Water regime (W) | 37.59 | 0.0002*** | 2.13 | 0.1618 | 1.66 | 0.2296 |
| Cropping system (C) | 3.05 | 0.0974 | 1.52 | 0.2453 | 0.35 | 0.7171 |
| W x C | 1.56 | 0.2613 | 0.75 | 0.4853 | 0.82 | 0.4720 |

**Table A4** PERMANOVA results (F-ratio, p-value, and R^2^) showing the effect of drought, cropping system, and soil layer on the prokaryotic and fungal β-diversity at wheat ripening. Differences are based on Bray-Curtis dissimilarities and are analysed for all soil layers combined and soil layers separately (i.e. 0-0.25 m, 0.25-0.5 m, 0.5-0.75 m). Heteroscedasticities are indicated as superscript ^1^. Values p < 0.05 are indicated in bold.

| Prokaryotes | | | | | | | | |
| --- | --- | --- | --- | --- | --- | --- | --- | --- |
|  | Soil layers combined | | 0-0.25 m | | 0.25-0.5 m | | 0.5-0.75 m | |
|  | F (p) | R^2^ | F (p) | R^2^ | F (p) | R^2^ | F (p) | R^2^ |
| Water regime (W) | 1.48 (0.1330) | 0.013 | **2.18 (0.0281)** | 0.068 | 1.04 (0.3735) | 0.044 | 0.84 (0.5801) | 0.038 |
| Cropping System (C) | **3.21 (0.0011**) | 0.057 | **5.05 (0.0001) ^1^** | 0.316 | **1.43 (0.0453**) | 0.122 | 0.71 (0.8854) | 0.064 |
| Soil layer (S) | **18.58 (0.001)** | 0.333 |  |  |  |  |  |  |
| W x C | 1.14 (0.2757) | 0.020 | 0.83 (0.6391) | 0.052 | 0.75 (0.8939) | 0.064 | 0.96 (0.4764) | 0.087 |
| W x S | 1.07 (0.3176) | 0.019 |  |  |  |  |  |  |
| C x S | 1.33 (0.1149) | 0.048 |  |  |  |  |  |  |
| W x C x S | 0.72 (0.8882) | 0.026 |  |  |  |  |  |  |
|  | | | | | | | | |
| Fungi | | | | | | | | |
|  | Soil layers combined | | 0-0.25 m | | 0.25-0.5 m | | 0.5-0.75 m | |
|  | F (p) | R^2^ | F (p) | R^2^ | F (p) | R^2^ | F (p) | R^2^ |
| Water regime (W) | **2.90 (0.0020)** | 0.031 | **5.62 (0.0001)** | 0.182 | 1.27 (0.0834) | 0.052 | **1.48 (0.0245**) | 0.063 |
| Cropping System (C) | **3.34 (0.0001)** | 0.071 | **3.87 (0.0001) ^1^** | 0.238 | **1.63 (0.0001)** | 0.134 | 1.08 (0.2779) | 0.091 |
| Soil layer (S) | **9.79 (0.0001) ^1^** | 0.208 |  |  |  |  |  |  |
| W x C | 1.78 (0.1852) | 0.025 | 0.91 (0.5984) | 0.056 | 0.86 (0.8589) | 0.071 | 0.98 (0.5186) | 0.083 |
| W x S | **1.60 (0.0268) ^1^** | 0.034 |  |  |  |  |  |  |
| C x S | 0.86 (0.7949) | 0.067 |  |  |  |  |  |  |
| W x C x S | 0.79 (0.9309) | 0.034 |  |  |  |  |  |  |

**Fig. A1** Mobile labelling chambers from stem elongation (1) to ripening (11). ^13^CO_2_ was applied with syringes as seen at the first timepoint (1). A portable CO_2_-analyzer (LI-COR, Lincoln, US) was used to measure CO_2_ concentration in the chambers (7).

**Fig. A2** Pictures of the microplot sampling. (a) pictures of the crown root of wheat as defined in this study at wheat ripening, (b) picture of the microplot with removed aboveground biomass and crown roots, sampling the first 0-0.25 m as whole monoliths, (c) sampling the intermediate (0.25-0.5 m) and deep (0.5-0.75 m) with a riverside auger between and withing the row.

**Fig. A3** Visual protocol of the root and rhizosphere processing for 0-0.25 m and 0.25-0.75 m.

**Fig. A4** Rarefaction curves for 71 fungal (a) and all 72 prokaryotic (b) samples in different sampling depth and cropping systems (i.e. BIODYN, biodynamic cropping system; CONFYM, mixed conventional system; and CONMIN, mineral fertilized conventional system). A sample with a low read count was excluded (one fungal sample).

**Fig. A5** Water regime and cropping system effects (i.e. BIODYN, biodynamic cropping system; CONFYM, mixed conventional system; and CONMIN, mineral fertilized conventional system) on relative rhizodeposition to total below-ground carbon in the three soil layers (i.e. 0-0.25 m, 0.25-0.5 m, and 0.5-0.75 m) in drought-induced and rainfed control plots. Means and standard errors are shown. Different letters indicate significant interaction (p < 0.05) assessed by least square means.

**Fig. A6** Spearman correlation analysis of rhizodeposition C and fine and total root C across the water regime and cropping systems (i.e. BIODYN, biodynamic cropping system; CONFYM, mixed conventional system; and CONMIN, mineral fertilized conventional system). Correlation of rhizodeposition C with a) fine root C in 0-0.25 m, b) with total root C in 0-0.25 m, c) fine root C in 0.25-0.5 m, d) total root C in 0.25-0.5 m, e) fine root C in 0.5-0.75 m, and f) total root C in 0.5-0.75 m. The correlation coefficient (rho) and p-value are provided.

**Fig. A7** Water regime and cropping system effects (i.e. BIODYN, biodynamic cropping system; CONFYM, mixed conventional system; and CONMIN, mineral fertilized conventional system) on microbial abundance, α-diversity, and oligotroph:copiotroph ratio in the three soil layers (i.e. 0-0.25 m, 0.25-0.5 m, and 0.5-0.75 m) in drought-induced and rainfed control plots. Prokaryotic abundance (a), fungal abundance (b), prokaryotic α-diversity (c), fungal α-diversity (d), and oligotroph:copiotroph ratio (e) measured in rhizosphere soil. Significant differences between the water regime (W), cropping system (C), and their interaction for each soil layer assessed by ANOVA are indicated as p < 0.06, * p < 0.05, ** p < 0.01, *** p < 0.001. Means and standard errors are shown.

**Fig. A8** Principal coordinate analysis (PCO) ordinations and canonical analysis of principal coordinates (CAP) of prokaryotic and fungal communities based on Bray-Curtis dissimilarities assessed with ASV abundances across all soil layers in the different water regimes and cropping systems (i.e. BIODYN, biodynamic cropping system; CONFYM, mixed conventional system; and CONMIN, mineral fertilized conventional system). (a) PCO of prokaryotic communities, (b) PCO of fungal communities, (c) CAP of prokaryotic communities, and (d) CAP of fungal communities.
